# Management of Symptomatic Hemorrhage From a Developmental Venous Anomaly

**DOI:** 10.7759/cureus.58130

**Published:** 2024-04-12

**Authors:** Matthew C Findlay, Robert B Kim, Angelica R Putnam, William T Couldwell

**Affiliations:** 1 Department of Neurosurgery, University of Utah, Salt Lake City, USA; 2 Department of Pathology, University of Utah, Salt Lake City, USA; 3 Department of Neurosurgery, University of Utah, Huntsman Cancer Institute, Salt Lake City, USA

**Keywords:** vascular malformation, symptomatic, hemorrhage, developmental venous anomaly, case report

## Abstract

Developmental venous anomalies (DVAs) are clinically benign, low-flow vascular malformations that classically hemorrhage only when associated with a cerebral cavernous malformation. It is very rare for an isolated DVA to hemorrhage. Resection of the DVA is generally contraindicated because of the high risk of venous infarct. We present the case of a large symptomatic hemorrhage stemming from an isolated DVA. The hematoma was evacuated and the hemorrhagic portion of the DVA was resected. This case demonstrates that in rare circumstances, careful resection can successfully treat hemorrhagic DVAs.

## Introduction

Developmental venous anomalies (DVAs) are common embryological vascular abnormalities estimated to exist in 2.6-6.4% of the general population [1,2]. These benign structures are almost always asymptomatic and function to drain cerebral venous blood. Because DVAs are most commonly clinically silent abnormalities, they are often discovered incidentally during brain imaging for other unrelated symptoms. DVAs rarely bleed in isolation, with an incidence of approximately 0.22-0.34% per year [3,4]. Most intracerebral hemorrhages involving DVAs are attributed to associated cerebral cavernous malformations (CCMs) rather than DVAs themselves [5,6]. In extremely rare cases, a DVA may hemorrhage and cause symptoms. Resection of DVAs is generally contraindicated because removing a large collecting venous system like a DVA is likely to lead to significant cerebral infarct [7]. Resecting a DVA is only considered in the presence of significant symptomatic hemorrhage, and if the malformation is located in an accessible, noneloquent region of the brain [1,8]. We report a case of a large symptomatic hemorrhage stemming from an isolated DVA. The hematoma was successfully evacuated and the hemorrhagic DVA channels safely resected.

## Case presentation

**Figure 1:**
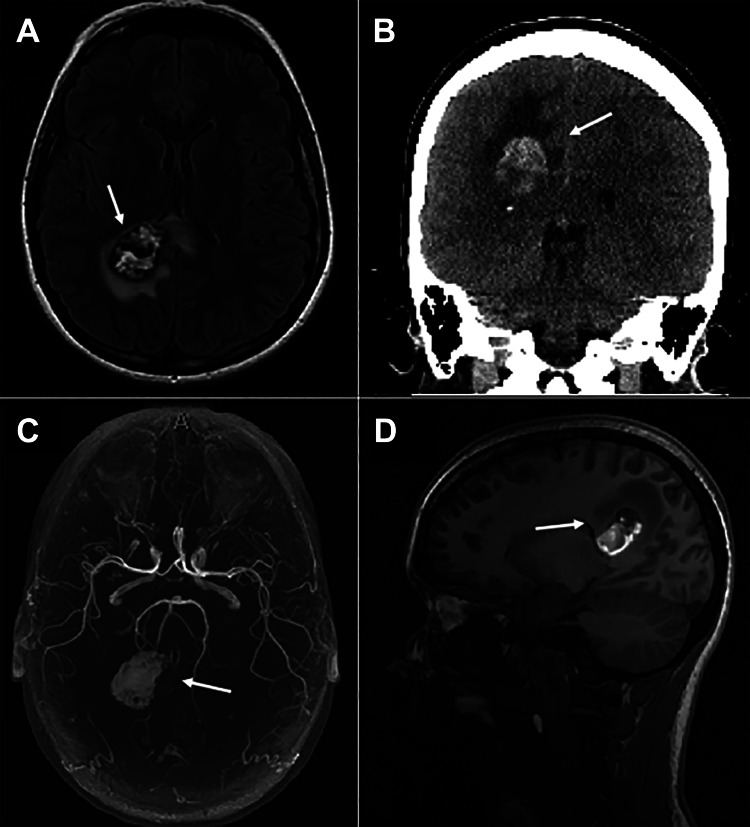
Magnetic resonance imaging of the enhancing mass. Preoperative (A) axial fluid-attenuated inversion recovery (FLAIR), (B) coronal CT, (C) axial 3D magnetic resonance angiogram, and (D) sagittal fast spoiled gradient echo (FSPGR) imaging obtained at presentation. The arrows in each image point to the large 3.0 × 2.5 cm hemorrhage near the occipital horn of the right lateral ventricle and the significant surrounding vasogenic edema.

A woman in her 20s with a one-month history of intractable headaches presented with the worst headache of her life. A computed tomography (CT) scan of the head revealed a heterogeneous 3.0 × 2.5 cm hemorrhage near the right occipital horn with surrounding vasogenic edema. Further imaging with brain magnetic resonance (MR) imaging and angiography demonstrated an enhancing, circular mass with gradient-echo susceptibility and a rim of diffusion restriction (Figure 1).

Investigations

Besides the severe headaches, the patient had normal neurological examination findings. She denied any changes in her vision, numbness, weakness, or any paresthesia. There were no constitutional symptoms. Her family history was negative for Osler-Weber-Rendu syndrome, CCM syndrome, or brain tumors. She denied any cigarette smoking or use of drugs/alcohol.

The imaging characteristics pointed initially toward hemorrhagic meningioma as a probable diagnosis, but other hemorrhagic neoplasms or vascular malformations, including CCM, could not be fully ruled out. The patient underwent a metastatic work-up, which was negative. After a long discussion of the available options with our team and her family, the patient elected surgical resection of the symptomatic, hemorrhagic mass lesion.

Treatment

**Figure 2:**
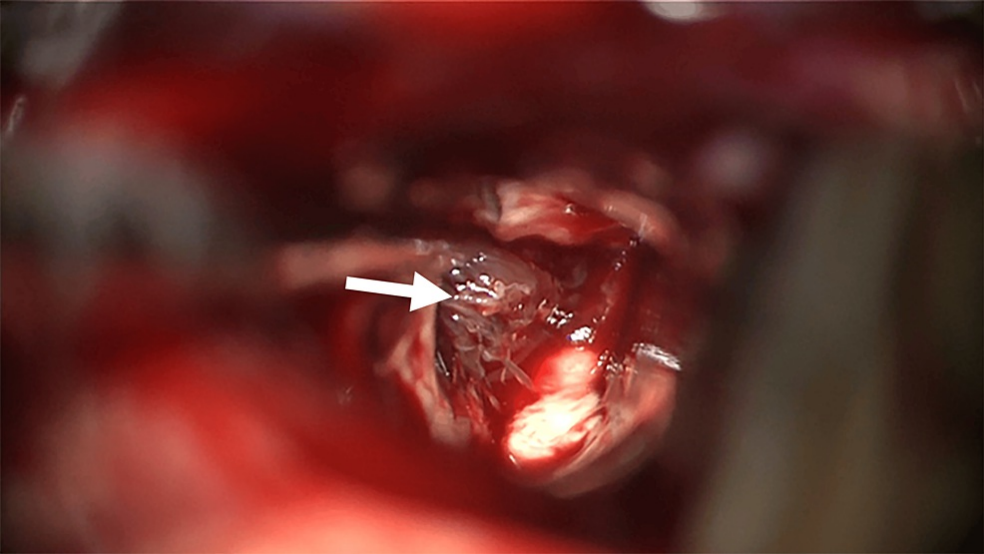
Intraoperative imaging showing the hemorrhage pocket and caput medusae coalescing onto the ruptured developmental venous anomaly (arrow).

The patient was placed in a semi-sitting position under general anesthesia. Rigid head fixation was achieved using the Mayfield skull clamp (Integra LifeSciences, Princeton, NJ), and then neuronavigation and intraoperative neuromonitoring were set up. The shortest possible trajectory to the target through the right parietal lobule was chosen, and a skin incision was planned just over it. A right parietal craniotomy was performed in a standard fashion, after which the dura was incised in a cruciate pattern. Again, using the predetermined trajectory, a corticectomy was made, and white matter was carefully dissected while preserving the cortical venous structures. The hemorrhage pocket and lesion were soon encountered (Figure 2). The lesion presented a gross appearance of CCM, although a primary tumor could not be initially ruled out. The hemorrhage was evacuated, and the hemorrhagic mass was excised circumferentially. The mass was noted to be in various stages of hemorrhage and contained thrombosed veins. Multiple specimens were sent for pathology review and were diagnosed as hemorrhagic DVA.

Outcome and follow-up

**Figure 3:**
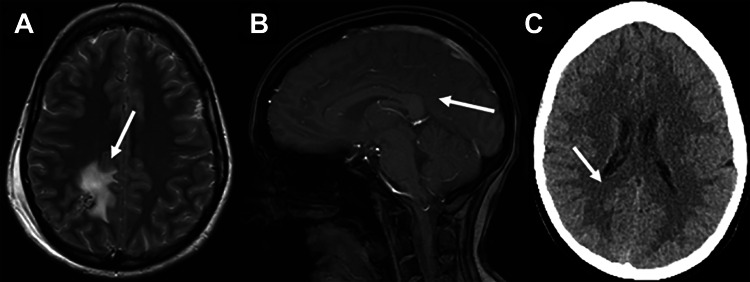
Postoperative images taken at 24 hours, three months, and nine months after surgery, respectively. A: Axial T2-weighted magnetic resonance (MR) imaging of the brain taken 24 hours postoperatively. Residual blood products (arrow) are visible. B: Sagittal MR imaging scan of the brain obtained three months postoperatively. Overall, a near-complete resolution of the T2 signal (arrow) that had been seen in the prior images is demonstrated. C: Noncontrast CT of the head obtained nine months postoperatively showing total resolution (arrow) of the lesion effect.

The patient tolerated the procedure well and was discharged on postoperative day four. At follow-up three weeks postoperatively, the patient reported her headaches were diminishing. Three months later, an MRI of the brain showed no evidence of further hemorrhage, and nine months later, a head computed tomography angiography also demonstrated no indication of additional hemorrhage (Figure 3). Eighteen months after the operation, the patient reports feeling nearly 100% recovered.

## Discussion

DVAs are abnormal vascular structures that did not develop properly in the embryo but serve nonetheless to drain normal venous return [9,10]. These abnormal vascular structures are the most common of cerebral vascular malformations, existing in an estimated 3% of the general population [7,9]. DVAs are usually asymptomatic and often found incidentally through unrelated radiographic imaging. Upon discovery, additional imaging, including CT, MRI, and digital subtraction angiography, is often warranted to optimize the radiographic characterization of the lesion, rule out other pathologies, and assess preoperative surgical feasibility [11]. Because these undeveloped vascular structures generally successfully drain blood from brain tissue, they are clinically benign and are almost always managed conservatively when encountered [7].

Morphologically, DVAs are characterized by a cluster of converging dilated veins that coalesce into a larger collecting vein [6]. This unique radiographic DVA appearance is termed caput medusae and closely resembles the radiating spokes on a bicycle wheel [8]. In our case, as seen in Figure 1, the caput medusae signal is not visible and was likely obscured by the large hemorrhage. However, the intraoperative image in Figure 2 shows the many tortuous and abnormal venous structures coalescing around the collecting vein.

**Figure 4:**
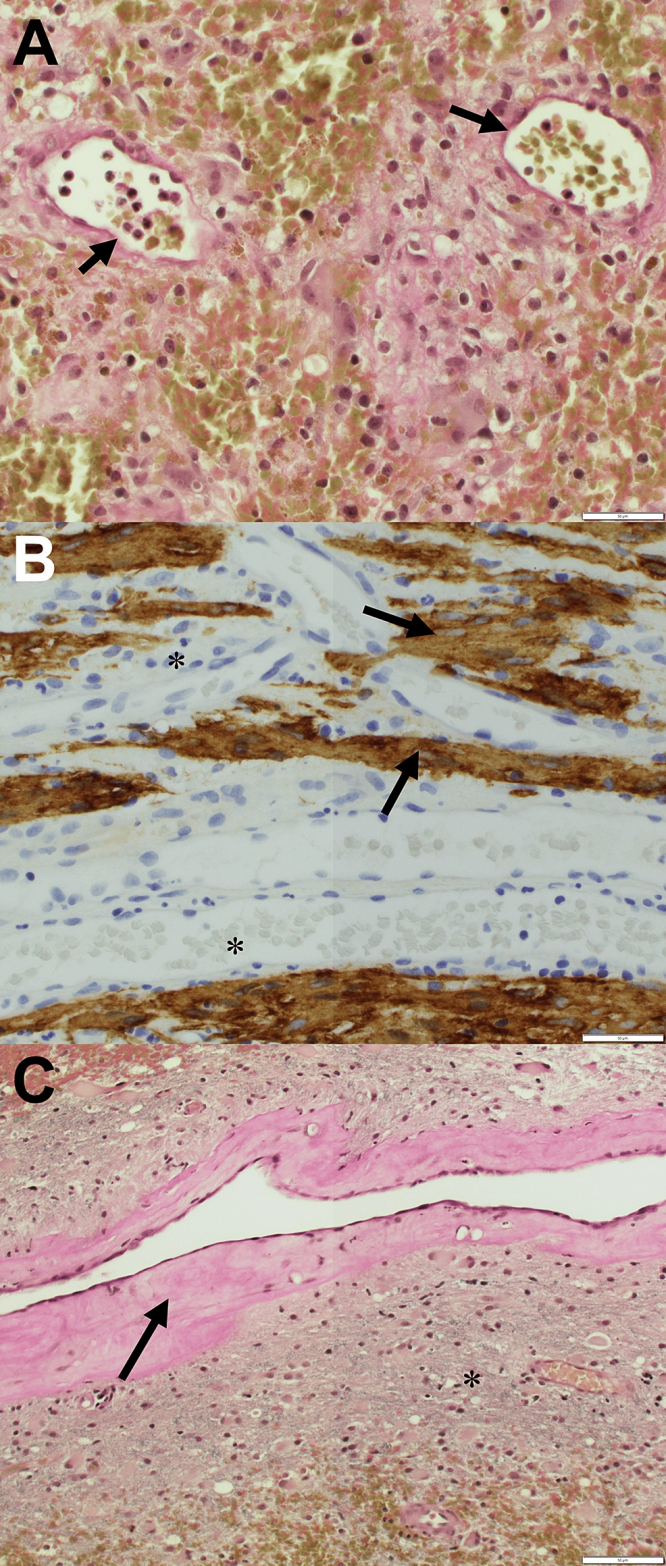
Histologic images from the excised developmental venous anomaly. A: Vessels (arrow) are thin-walled and do not contain an inner elastic lamina, which is confirmed with an elastic stain. B: Glial fibrillary acidic protein (GFAP) immunohistochemical stain demonstrates ample intervening neural parenchyma (arrow) between vascular structures (star). C: A prominently hyalinized vessel wall (arrow) is seen on the hematoxylin and eosin stain. The adjacent neural parenchyma (*) is well-preserved.

Histologically, DVAs are distinguished from primary tumors with their network of hyalinized veins with intervening neural parenchyma [12]. DVAs are also characterized by a lack of smooth muscle and elastic tissue [1,5]. Microscopic examination of our excised lesion revealed a vascular malformation composed of large, tortuous, thrombosed vessels demonstrating evidence of acute and chronic hemorrhage. Some vasculature in our specimen was hyalinized while the majority was not (Figure 4). However, clear intervening neuropil was confirmed using glial fibrillary acidic protein immunostaining. Intervening neural tissue denoted by neuropil is not found in CCMs [13]. Elastic staining of our sample showed a lack of internal elastic lamina as would be expected in venous structures [10]. Pertinent histological negatives that helped rule out arteriovenous malformation (AVM) as a diagnosis included the well-preserved organization of the surrounding parenchyma and lack of both venous adventitial fibrosis or arterial components [14]. As a whole, these histological findings confirmed the identity of our resected lesion as a DVA and ruled out meningioma, CCM, isolated AVM, and other possible vascular malformations.

Intracerebral hemorrhage is an acutely dangerous condition that can lead to seizures and permanent focal neurological deficits. When DVAs are found associated with symptomatic intracerebral hemorrhage, it is almost always due to a nearby hemorrhagic CCM [1]. Interestingly, DVAs have been indicated to play a causal role in the growth and development of CCMs [15]. DVA-induced CCM pathogenesis is hypothesized to occur as chronically elevated venous pressure causes microhemorrhages around the DVA leading to angiogenic proliferation and eventual CCM formation [15]. CCMs are characterized by a distinct popcorn-like appearance on brain imaging and are composed morphologically of cavernous capillaries [16]. When a CCM does symptomatically hemorrhage in a noneloquent location, lesion resection is the current gold-standard treatment [17]. In our case, no cavernoma was encountered intraoperatively, radiographically, or histologically.

Surgical removal of DVAs is classically contraindicated because they assist in normal venous drainage despite their anomalous vasculature and catastrophic venous infarction can result following DVA resection [5,8]. Even when DVAs do spontaneously hemorrhage, evacuating the hematoma while leaving the DVA intact is the most preferred course of action [7]. Only in cases of surgically accessible DVA causing a symptomatic hemorrhage that shows evidence of previous hemorrhage should resection be considered [5]. In our case, the rapid onset of severe symptoms and the dangerous size of the hemorrhage, in addition to the evidence of previous chronic bleeds, prompted resection of the hemorrhaging DVA with careful preservation of non-hemorrhagic venous structures. Although our preoperative assessment and the urgent clinical context prompted surgical treatment for a presumed hemorrhagic meningioma, additional preoperative DSA imaging would have been useful to better exclude AVM, and we support the literature wherein such a workup is recommended [18,19]. Postoperatively, there was no evidence of infarction and over one year postoperatively, the patient reports feeling nearly 100% recovered.

In terms of pathophysiology, hemorrhagic DVAs are caused by either an imbalance of blood inflow and outflow or mechanical compression [20]. AVMs and other vascular abnormalities can create an increased inflow of blood to the DVA that can cause downstream DVA hemorrhage. Similarly, diminished outflow tracts such as thrombosis in the draining vein of the DVA can cause intracerebral hemorrhage [7,12]. Such blockage increases pressure in the DVA feeder vasculature, leading to hemorrhage [12]. The sample we resected contained thrombosed veins, so this represents a plausible mechanism for the hemorrhagic presentation in our case. The other accepted mechanism for DVA hemorrhage is mechanical compression. In these cases, conditions such as obstructive hydrocephalus or neurovascular nerve compression syndromes exert physical force on the DVA leading to hemorrhage [7,10,20]. We did not observe any evidence of mechanical compression in our case.

## Conclusions

In this report, we described the case of a young woman experiencing intractable headaches who presented with a large symptomatic hemorrhage exhibiting mass effect. Surgical exploration allowed us to evacuate the hematoma. Because of the large hemorrhage combined with evidence of previous bleeds, the hemorrhagic mass was excised. A composite analysis of the radiographical imaging, intraoperative evaluation, and histological analysis indicated with a high degree of certainty that the excised mass represented a DVA. The patient experienced no venous infarct postoperatively, and her symptoms have greatly diminished with her reporting a near-full recovery. This case demonstrates that in rare circumstances, careful resection can be employed to treat hemorrhagic DVAs.
